# “It’s being part of the big picture, even though you’re a tiny jigsaw piece”—motivations and expectations of individuals participating in the Enroll-HD observational study

**DOI:** 10.1007/s12687-020-00459-3

**Published:** 2020-03-10

**Authors:** E. Davies, D. Craufurd, R. MacLeod

**Affiliations:** 1 Division of Evolution and Genomic Sciences, School of Biological Science, University of Manchester, Manchester, UK; 2 Department of Clinical Genetics, Addenbrooke’s Hospital, Hills Road, Cambridge, UK; 3 Manchester Centre for Genomic Medicine, St Mary’s Hospital, Manchester Academic Health Science Centre, Manchester University Hospitals NHS Foundation Trust, Manchester, UK

**Keywords:** Huntington’s disease, Research participation, Enroll-HD

## Abstract

Predictive test guidelines for Huntington’s disease (HD) recommend individuals are offered opportunities to participate in research regardless of test outcome. Consistent with most HD centres of excellence, the Manchester Centre for Genomic Medicine (MCGM) invites eligible individuals to participate in the observational study, Enroll-HD. Limited research has been conducted to date on the views of research participants and the possible impact of participation. The aim of this qualitative study was to explore the experiences of ten individuals taking part in the Enroll-HD study following pre-symptomatic testing for HD. Half of the individuals had tested positive for the HD mutation and the other half had tested negative. Participants were generally motivated to take part in the study by both personal and altruistic reasons. Overall, they were very positive about participation in Enroll-HD. Valuable aspects included good relationships with the research/clinical team, increased understanding of the condition, an enhanced self-image and a shared experience with affected parents. Issues for improvement to encourage participation included access to study site and more regular communication about study progress. Participants, while generally optimistic about research progress, were realistic about challenges.

## Introduction

### Overview of Huntington’s disease

Huntington’s disease (HD) is a rare progressive neurodegenerative disorder in which movement, behaviour and cognition are affected (Huntington 1872; Craufurd et al. 2015). It affects approximately 3 individuals per 100,000 worldwide, with a higher prevalence of 10 per 100,000 in European populations (Harper 1992; Pringsheim et al. 2012). It is a monogenic disorder that is autosomal dominantly inherited (Huntington 1872; Craufurd et al. 2015) and is caused by an expansion of a CAG trinucleotide repeat in the gene huntingtin (HTT) (MacDonald et al. 1993).

The genetic test for HD measures the length of the CAG repeat in the HTT gene, with a “positive” result meaning the individual carries a pathogenic allele with 39 or more CAG repeats (Novak and Tabrizi 2010). Genetic testing can have one of three main purposes: diagnostic, predictive or prenatal testing (Myers 2004). Predictive testing is carried out in a person who has no symptoms but is at risk of developing HD due to their family history. A mutation-positive result indicates that the person will develop HD at some point in the future but gives no information on which symptoms will present and when. Approximately 15–26% of people at risk decide to undergo predictive testing (Quarrell et al. 2014) and do so for several reasons, including wanting to relieve uncertainty and better prepare for the future (Evers-Kiebooms and Decruyenaere 1998; Tibben 2007).

While the genetic cause and nature of HD have long been identified, there are currently no disease-modifying treatments available. Patient care is currently focused on the management of symptoms to improve quality of life. This can include medication and surveillance to counselling and therapy and will often change as the condition progresses. Research for better treatments and a successful cure is on-going. Several different pharmacologic agents are being investigated (Ross and Tabrizi 2011) and disease-modifying trials, making use of gene silencing technology, have recently been launched (Matsui and Corey 2012). Finally, through major international studies such as TRACK-HD, PREDICT-HD and Enroll-HD, HD research has been elevated to a global stage and there are hopes for an improved understanding of the condition and how it affects people (93).

### Participation in research

The progression of genetic research into conditions such as HD relies quite heavily on the involvement of human participants. This allows for service improvements to be made, different drug targets to be uncovered and new treatments to be trialled and utilised. In terms of genetic research, scientists can better understand human genetic variation and the genetic mechanisms of conditions such as HD. Furthermore, with the current pace of medical and genomic research, the number of clinical studies and thus the demand for human participants are rapidly increasing (Sung et al. 2003).

With such an importance placed on recruiting human participants for genetic research studies, there has been some research conducted on human subject participation. Most of this has focused on issues of informed consent and the storage of biosamples and personal data (McGuire and Beskow 2010; Wang et al. 2001). Some research has also been conducted into what motivates people to take part and any barriers that may discourage them. One of the primary motivators found for wanting to take part in research is altruism. Studies have found that many patients are keen to help advance science, assist the medical researchers and potentially help future patients (Kost et al. 2011; Facio et al. 2011). However, some studies have found that altruistic intentions are often tempered by other motivations (Nappo et al. 2013; Canvin and Jacoby 2006; McCann et al. 2010). Given the genetic nature of certain conditions, in some cases, individuals are participating in the hopes of benefiting their relatives (Hallowell et al. 2010). Several studies have also found that many participants are driven to take part because of some form of personal gain (Grant 2015; Tishler and Bartholomae 2002). Participating could also be a way of gaining more information about their condition (Donoghue et al. 2014; Treloar et al. 2007) or provide an opportunity to talk with others similarly affected (Trottier et al. 2013). Others may be participating in the hopes of receiving better medical care, through increased access to medical professionals and a more regular assessment of their condition or by trialling new, potentially effective, therapeutics (Luchtenberg et al. 2015). Researchers have also found that even interview-based participation can have some therapeutic benefit for patients (Shamai 2003). Indeed, some studies have found that taking part in research gives some participants an enhanced self-image and feelings of doing good (Michie et al. 2011).

Research into participation provides helpful insights into why some people choose to take part and others do not. This may ultimately help to improve recruitment and meet the increasing demand for human participants while also making research studies beneficial for all involved. However, as each study is unique, generalisations can only be made to a certain extent. In order to optimise each individual study, it is important to gain the perspectives and opinions of those involved.

### Enroll-HD

Enroll-HD is currently the world’s largest Huntington’s disease observational study. It is designed to monitor the condition and how it presents and changes over time (93). It has been cited as a “resource for the entire HD community, including patients, families, patient advocates, clinicians and other healthcare professionals, researchers, and anyone else who has a connection to HD” (93). Enroll-HD builds upon two existing HD registries: the COHORT study based in North America and Australia, and the REGISTRY study from Europe, to create a much larger database for HD research. It currently has over 17,000 participants across 19 different countries and hopes to eventually incorporate over 20,000 people (93). It is a prospective open-ended study, allowing the condition to be tracked over time rather than relying on participants’ recollection of their symptoms and how they have changed.

**Fig. 1:**
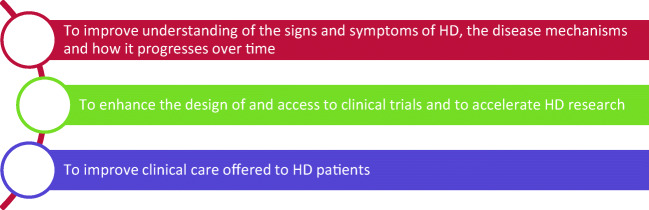
Main aims of Enroll-HD observational study (94)

**Fig. 2:**
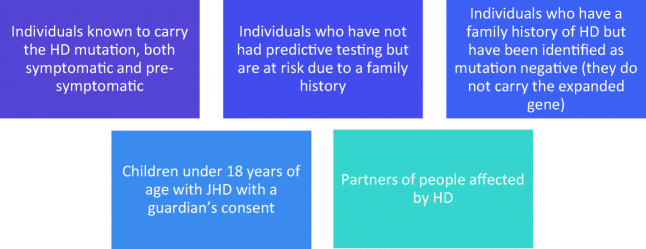
Groups eligible for Enroll-HD participation

With the overarching aim of improving knowledge of HD and enabling clinical research, those conducting Enroll-HD have stated three main objectives they hope the study will achieve (Fig. 1):

There are several different groups of people eligible to participate in Enroll-HD (Fig. 2). Once a part of Enroll-HD, participants attend annual study visits for a series of assessments. These are designed to examine the various aspects of the condition as well as the functional status of the individual and their current quality of life (94). Researchers wanting to conduct HD-related studies can then access data and biosamples collected from these assessments.

As part of the recruitment process, the study has posed some possible reasons people may have for wanting to participate. These include the possibility of helping to improve HD research, find new and effective ways to treat the condition, meeting healthcare professionals and learning more about the condition and any studies and trials in which they may want to become involved. They also suggest that being involved in clinical research can be “gratifying because it is a chance to take action” and that participants can feel empowered as it allows them to “regain some control over the disease” (95). It has also been suggested it could act as a coping strategy for some, or that certain groups such as the pre-symptomatic gene mutation carriers can gain access to more regular medical attention (95). This study adds to the literature by exploring how people come to be involved in Enroll-HD and their experiences of participation.

## Methods

This qualitative study used semi-structured telephone interviews to explore the thoughts and opinions of ten individuals participating in the Enroll-HD study post predictive testing. Ethical approval for this study was sought and granted by the NHS Research Ethics Committee (REC reference: 15/NW/0013. IRAS project ID: 134748).

There were 3 main research questions:

- What reasons do HD mutation carriers and those with a negative gene mutation status have for wanting to take part in Enroll-HD?
- What expectations do these individuals have for being involved in this study?
- How do these individuals find the process of participating in Enroll-HD?

### Participants

Purposive sampling was used to identify eligible participants: current Enroll-HD participants, 18 years or older, with a mutation-positive or -negative status established. They were recruited through the Manchester Centre for Genomic Medicine (MCGM) Enroll-HD register and their patient files were checked to ensure eligibility and suitability for this project. A sample size of ten was chosen, to allow for any similarities and differences between data sets to be examined, while still ensuring a thorough qualitative analysis of each interview. Taking into account the predicted uptake, invitation packs including consent forms were sent out to 12 prospective participants that fit the eligibility criteria. As part of the Enroll-HD recruitment process, these participants had previously consented to being approached to take part in other related studies.

### Data collection

From the 12 invitation packs sent out, 10 consent forms were returned, 5 from gene mutation–positive individuals and 5 from gene mutation–negative individuals. These participants were then invited to participate in a telephone interview. A semi-structured interview style was utilised, with the interviewer following a flexible interview guide. This ensured all important points were addressed, while also encouraging the participant to discuss their opinions freely. Where further information or clarification was required, further prompts were introduced. Each participant was told they could pause or stop the interview at any time they wished and were reminded that if they had any concerns or issues they could contact the Genomic Medicine department at St Mary’s Hospital, Manchester. The interviews were recorded, transcribed verbatim and anonymised upon completion.

### Data analysis

A thematic analysis was used to explore the experiences of participants and to identify and analyse any themes that emerged (Braun and Clarke 2006). The transcripts were first read individually several times before initial codes and key phrases were annotated. Groups of codes were then gradually transformed into representative subthemes, with those arising from each interview compared before being formulated into a table of subthemes. This analysis was performed in a cyclical manner and the original transcripts were frequently referred back to throughout the analysis to check emerging subthemes accurately represented the data. These subthemes were then organised into overarching themes to allow for connections to be formed across all the transcripts. In order to reduce researcher bias and to ensure the narrative account accurately reflected the data, the research supervisor also analysed the themes and subthemes collected.

## Results

Ten individuals participated in the study (Table 1). As requested, all had undergone HD predictive testing, with some having had testing many years ago and others much more recently. Nine of the interviewees have first-hand experience of HD but not all had previous research experience. All attend their Enroll-HD assessments in the MCGM, with some recruited recently and others having participated for several years. All participants were recruited through genetic counselling and three had previously been involved in the PREDICT-HD study.

**Table 1:** Demographics of participants

Participant	Sex	Genetic status	Parent affected	Attends assessment with	Previous research experience	First-hand disease experience
1	M	Positive	Mother	Partner	No	Yes
2	F	Positive	Father	Partner	Yes	Yes
3	M	Positive	Mother	Alone	Yes	Yes
4	M	Positive	Mother	Partner	No	Yes
5	F	Positive	Mother	Partner	Yes	Yes
6	M	Negative	Father	Alone	Yes	Yes
7	F	Negative	Mother	Alone	No	Yes
8	F	Negative	Mother	Alone	No	Yes
9	F	Negative	Father	Alone	No	No
10	F	Negative	Father	Alone	Yes	Yes

Four main themes arose from the thematic analysis of the data: motivations for participation, experiences of participation, impact of participation and participant hopes and expectations.

### Motivations for participation

All participants commented on their main reasons for taking part in the Enroll-HD study. These could be clearly split between motivations participants perceived as being personal or “selfish” and altruistic motivations, with several individuals citing both as contributing to their involvement.

Some participants seemed to struggle with the idea that their motivations for taking part in HD research could be considered “selfish”. Several of the mutation-positive participants viewed the Enroll-HD visits as part of their general HD assessments and a way of potentially accessing clinical trials. For one participant who tested mutation positive, the hope was not so much for himself but rather in relation to his own children in the future. Some of the mutation-negative participants also reflected on their motivations for becoming involved in Enroll-HD as personal. Although they will not directly benefit from clinical trials, three participants had untested siblings and were concerned that they may develop the condition in the future. One participant seemed particularly motivated to stay involved with the study as this gave her access to the contact details of relevant health professionals, should her siblings wish to be tested in the future.

They’re both aware and I’ve always made it clear to them that if ever they changed their mind then I’ve got details of how to get in touch with people who could make that happen. (Female, mutation negative)

For another participant who tested negative, some of the motivation to take part was rooted in an interest in the science and research.

From a selfish point of view…I still have a physiological head, so I still like listening to that side of things and learning about what’s going on and seeing how things are developing. (Male, mutation negative)

While many hoped for personal benefits to participating, several participants were also involved for more altruistic reasons, wanting to help others and the wider HD community.

To put something into the mix and hopefully somehow, altogether, will help them to come up with a cure or… ways to slow things down or certainly help people… that was my main drive for doing it really. Even if it doesn’t help me directly, it will help other people in the future hopefully. (Male, mutation positive)

Several participants appreciated the need for willing volunteers in research; however, not all had previous research experience. For a few participants, there was also a clear feeling of being part of something bigger and wanting to contribute in a small way to further global research.

It’s being part of the big picture, even though you’re a tiny jigsaw piece, hopefully it helps others. (Male, mutation positive)

Several participants gave both “selfish” and altruistic reasons for participating. They described how while naturally they want to help themselves and those closest to them, they equally want to help strangers affected by the condition.

I suppose initially if you’re being selfish about it then it’s your own family first but certainly helping other people as well. If you see how people are with this condition, it’s not pleasant. (Female, mutation negative)

This awareness of the serious nature of the condition also appeared to contribute towards a desire to participate for several individuals. All but one of the participants had personal experience of HD through witnessing an affected parent. However, even the participant who had tested mutation negative and who had no first-hand experience gave the nature of the condition as a factor in her drive to stay involved in the study. As well as an awareness of the condition, for several of the mutation-negative participants, the feeling of being “the lucky one” also contributed to their desire to take part. They were aware that it had been equally likely that they inherited the mutated copy of the gene and wanted to help those who had not been as fortunate.

I wanted to help because I felt really lucky that I hadn’t got it and… if there was anything I could do to try and help, I was really happy to do that. (Female, mutation negative)

### Experiences of participation

All participants were generally very positive about their experiences taking part in the Enroll-HD study. All stated that they would recommend other eligible candidates become involved and all were willing to participate in further trials if asked.

Several of the participants commented on the clarity and availability of information about the study. Overall, they praised the recruitment process, with several describing how they received clear and thorough information without feeling pressured into participation. However, for a couple of participants, there was some confusion over which study they were now involved in. This was mainly a result of them having previously been involved in the PREDICT-HD study, the predecessor of Enroll-HD. Furthermore, since the recruitment process, some participants felt they had not received much information about the study or about any progress made. Several showed a significant interest in wanting to know more but seemed unaware of how they could access any study updates.

I’ll be quite honest; I don’t know that much about it. There seems to be stuff happening but I don’t think my mum’s ever been contacted to take part in any trials or anything. So I’m sort of thinking maybe there’s nothing. So I don’t know, I don’t know a massive amount. I would be interested to know more actually. (Female, mutation negative)

An aspect of Enroll-HD valued by almost all of the individuals interviewed was being able to see the same members of staff at each assessment.

…they kind of build up a picture of you as well and they know you, your characteristics and your personality. I realise that they see hundreds of people over the course of a year but it doesn’t take them long to say this has changed or this hasn’t so it’s nice really. (Male, mutation positive)

One participant discussed how she now travels a great distance just to remain with the same team that know her and her family.

I live in (undisclosed location) now so it’s a distance to travel but I’d rather stay in Manchester and everything. Grandad was treated there and so was Dad so all the doctors know the family so it makes it easier. …they know us so it just makes you feel more comfortable. (Female, mutation positive—note: location altered for anonymity)

Others discussed how seeing a familiar team gave them an opportunity to talk to people who are well informed as well as outside of their own family.

When asked about talking to family and friends, many of the participants reported only sharing their involvement in Enroll-HD with a select couple of people. For a couple of the mutation-negative participants, this seemed to be because of some family members not quite understanding why they were participating at all, given their genetic status. However, even some of the mutation-positive participants only shared their Enroll-HD participation with a partner or a single close relative.

All five of the mutation-negative participants attend their appointments alone; however, all but one of the mutation-positive participants attend with their partner. For the one participant who tested mutation positive and who attends alone, it was not because he did not want his partner there but more because his wife has not yet come to terms with the condition.

I come by myself. As I have done with all the visits and scans and everything really. My wife is not really…how can I say… she’s taken it hard really. Kind of just waiting for her kind of come round or be able to discuss the notion of something being wrong. (Male, mutation positive)

Four participants who tested mutation positive acknowledged how important their partners are and how involved and invested they are in the study.

My partner keeps up to date more than I do. On my yearly appointments I just turn up really but my partner is always looking out for research and everything else. (Male, mutation positive)

For a couple of participants, being involved in Enroll-HD has also allowed them to share experiences with their affected parent. This was reported by one of the participants who tested mutation positive and who was able to go through the process with her father. One of the mutation-negative participants also found that becoming involved in Enroll-HD gave her a better appreciation of what her affected mother experiences. This seems to allow for improved communication and understanding and helps her feel more connected to her mother.

I like telling mum I’ve had it done and she seems really positive that I’m taking part. …emotionally, it’s nice to just be able to share it with my mum. …She doesn’t talk quite as much as she used to but she’s very positive about it and I think she can see, like I say, it’s sharing something. Because my dad doesn’t do it and my brother doesn’t do it so we’ve got something that we share together really… I think she feels she can talk about it because I’ve experienced what she’s experienced. (Female, mutation negative)

Finally, for some participants, a few practical issues came with attending their annual visits. These included navigating the geography of the hospital and the parking facilities, giving blood and attending assessments on weekdays vs weekends. However, what was clear from these individuals was that this burden did not deter them from participating. Indeed, several participants, particularly the mutation-negative candidates, were willing to accept a minor hardship to continue contributing to the study.

Well the only [disadvantage] is going into Manchester, but that’s a physical thing, driving into Manchester but I can get over that. (Female, mutation negative)

One participant even described how he felt uncomfortable receiving expenses as he felt the money would be better spent on the research.

I’ll tell you what always makes me feel strange is when they (research team) try to make me take travel expenses. I just think spend the money on something else. You know, I don’t need my parking and my petrol to come and help. (Male, mutation negative)

### Impact of participation

While discussing their involvement in Enroll-HD, participants also alluded to the impact, if any, participation has had. As well as being satisfied with their Enroll-HD assessments, several participants also reported feeling better afterwards. For some of the mutation-positive participants, attending the visits was a way of being proactive and could often assuage anxieties they had about their condition. For some of the mutation-negative participants, this good feeling came as a result of knowing that their actions had potentially helped others.

It just makes you feel better; in terms of thinking you’re actually doing something. (Female, mutation positive)

It’s just nice to be involved in something. You know, you’re having some form of impact, its good. (Male, mutation negative)

For many of the participants, the Enroll-HD study does not appear to cause them any concern or to have a negative impact on their lives.

I don’t even think about it! Oh dear, that sounds awful, doesn’t it? I don’t even think about it, I just look in my diary....I’ve been and then I don’t think about it again. (Female, mutation negative)

For a couple of participants, while they were not worried about the visits themselves, they felt like their family were more anxious and wanted the assessments to go well.

For some sort of odd reason, the family want it to go well whereas it doesn’t cause me any anxiety or any worry. It’s definitely not a problem at all. (Male, mutation positive)

For all individuals regardless of test outcome, involvement in Enroll-HD seemed to increase awareness and understanding of Huntington’s disease. One participant who tested mutation positive also described how becoming involved in the study has helped his partner understand and accept the condition more.

Both myself and my partner have always come to the sessions together…I’ve sort of found dealing with the whole HD thing, I’ve kind of accepted it now and stuff but she hasn’t really and the process of going every 12 months does help her come to terms with it a little bit more. (Male, mutation positive)

As well as an improved understanding, for one mutation-positive participant, the most recent assessment also seemed to make them more aware of the reality of the condition.

I just think after this [visit], when they referred me for some tablets and they said they’ve noticed some very minute things in my movement and I think ‘is that it then?’ and I think after that, the penny has dropped that the likelihood is that this is my twilight of my life. (Male, mutation positive)

For this same participant, attending with his partner and hearing her observations of his symptoms also proved difficult, as it was not something of which he was previously aware.

The first one weren’t too bad, it was just after my test. But this one, I struggled a bit afterwards for about a week I think, just because my moods can change sometimes and… just listening to my partner telling them about how my moods can change, that was difficult to take. (Male, mutation positive)

A participant who tested mutation negative also found that the assessment brings the condition to the forefront of their mind, making them more concerned about their untested siblings and the reality of the situation.

For some of the participants, involvement in the Enroll-HD study also seemed to evoke a realisation that, while the assessments were going well now, things could be different. For the mutation-positive participants, this was an awareness of a future self, anticipating how their assessments would be as they become symptomatic.

I think what I’m more worried about is when I do my test next year they’ll see a difference, that’s what I’m worried about, that I’m not the same as I was last year. With all my movements and that, things can be different and that’ll get me thinking ‘is that the gene starting’. (Male, mutation positive)

For the mutation-negative participants, this appeared to be an awareness of an alternative self, of how things could have been if they had inherited the gene mutation.

For me, it’s easier because I know I haven’t got it. If I was doing the research and hadn’t been tested, I’d be absolutely terrified (Female, mutation negative)

### Hopes and expectations

All the participants interviewed were clear about their hopes and expectations for the study. Many participants hoped that the study would result in progress in HD research and treatment options. Several hoped there would one day be a cure for the condition but many also hoped for medications that could help better manage the symptoms.

It would be nice to see more on something that can slow the progress of it….I mean it would be best obviously to find a cure but to find something to slow it down and give someone quality of life for a longer time would be a lot better. (Female, mutation negative)

…if they can crack it, as it were, then that’d be wonderful but in the meantime if things can be made easier for people…then I’d be delighted. I’ve no real personal expectations for myself. I just hope it helps someone else. (Female, mutation negative)

Generally, participants were quite realistic in their expectations for the study and anticipated progress would happen over several years rather than overnight.

I realise that this is very much an initial stage so you know, I’m very much aware that we are, that the medical side of it is very much about trying to treat the symptoms at the moment and that there isn’t going to be some miracle cure next year, or the year after. And that research and everything else takes a long time. I’m very aware of all that. I guess my expectations are that hopefully in 30 years’ time I’ve played my part in a massive longitudinal study that has an impact. (Male, mutation negative)

Hoping we can find a cure, maybe not in my lifetime but certainly as my kids get older. (Male, mutation positive)

Despite this realistic attitude, participants were generally also quite optimistic about the outlook for individuals with HD and the progress in research. For one participant, in particular, this optimism was reinforced by an awareness of how things used to be for those affected by HD and the progress already made.

…to me, such strides have been made. From someone who was just shut away and there was no real knowledge, saying you’re loopy or whatever, for it be recognised as an actual condition… we are making such strides in so many areas that yes I do feel positive for the future. (Female, mutation negative)

## Discussion

### Motivations for participation

There has been quite extensive work into the reasons people give for engaging in clinical research. In this study, motivations were generally a combination of wanting to benefit personally and also wanting to help research and the wider community. Several of the participants seemed to struggle with admitting to wanting some form of personal gain and often described these motivations as “selfish”. However, many previous studies have found this to be a common motive for taking part in research. Others also seem to view research as a way of being proactive, of accessing more regular medical attention or to regain some control over their diagnosis (Trottier et al. 2013; Luchtenberg et al. 2015; Nappo et al. 2013). Several other studies also found people were motivated to participate to help relatives (Hallowell et al. 2010). A frequently cited personal motivation in the literature is gaining a financial reward (Grant 2015; Nappo et al. 2013); with several studies relying on monetary incentives to recruit healthy volunteers (Tishler and Bartholomae 2002). However, this was not the case in this study, with some mutation-negative individuals even willing to accept a minor financial hardship to attend their assessments. This difference is potentially due to the familial nature of HD. Unlike in non-genetic research, control subjects will frequently have significant personal experience and, due to at-risk relatives, possibly a heightened investment in the research. As a consequence, personal, or “selfish”, motivations are perhaps inevitable and financial incentives less necessary. However, altruism was another motivation cited by many of the participants and there is growing literature that supports this (Kost et al. 2011; Facio et al. 2011). This altruistic attitude was reinforced by the participants’ awareness of the reality of HD. This awareness and lived experience of a condition as a contributing factor in participation has also been previously observed (Treloar et al. 2007). Furthermore, solidarity with others who are affected and with future generations at risk has been observed in a study examining young people’s motivations for participating in clinical trials (Luchtenberg et al. 2015). Almost all participants cited both altruistic and “selfish” reasons as contributing to their involvement in research, echoing the work of Hallowell et al. (2010). They too found that often motivations are interdependent and viewing them in isolation is insufficient when attempting to understand why people participate in research. For some of the participants in this study, altruism was one of their primary motivations; however, for others, it was more of an incidental advantage. This has previously been described as “weak altruism” (Canvin and Jacoby 2006) or “conditional altruism” (McCann et al. 2010) and indeed, Hallowell et al. (2010) concluded that purely altruistic participation is likely rare. As mentioned, it is perhaps inevitable having personal motivations in genetic research and some have argued that appealing to altruism alone is insufficient when attempting to recruit research participants (Hunter et al. 2012). However, the claim of altruism has a clear benefit for some of the Enroll-HD participants. Several reported feeling good about themselves and their contribution to the study. Indeed, this enhanced self-image is a well-recognised effect of altruistic acts (Post 2005) and as Michie et al. conclude, pride in having contributed to society can itself be a motivator (Michie et al. 2011). While it may be secondary for some, it may also encourage long-term involvement.

### Experiences of participation

Participants were overall very positive about the experience of taking part in research highlighting, in particular, the relationship with the research team. In comparison to genetic counselling research where the value of the counselling relationship is well documented (McAllister et al. 2008), the significance of this relationship in a research setting has received less attention. However, a small number of other studies have also noted the value research participants attribute to having a positive relationship with the research team (Trottier et al. 2013; Kost et al. 2011). Having a familiar, knowledgeable team can be valuable in several ways. For some participants, it was comforting to see the same people each time and gave them a safe space to talk about themselves and the condition. Indeed, this therapeutic benefit has been shown to occur in interview-based research (Shamai 2003). Others have found that a positive relationship with the staff was one of the main reasons participants gave for their continued involvement in research (Kost et al. 2011). Importantly, the Enroll-HD study also highlights the benefits of combined clinical and research clinics. The consistency and quality of the relationship with the clinician within the Enroll-HD setting was an important aspect of research involvement for several participants.

Many of the participants in this study only shared their research involvement with one or two close relatives, most often their partner. This would be an interesting area for further investigation. The importance of partners was clearly highlighted by many participants. The role and influence of companions have previously been established in medical consultations but not in a research setting (Andrades et al. 2013). This study found that partners were often more invested in the research than the participants were, supporting evidence from the literature that suggests partners will often actively gather information about the condition as an initial coping strategy (Kessler 1993). Despite their direct involvement, partners have frequently been referred to as the “forgotten persons” in an HD family (Kessler 1993). Evidence suggests they can often experience the same, if not higher levels of psychological distress after the predictive test result but will seek much less social support than their affected spouses (Decruyenaere et al. 2005). This study suggests that the psychological benefits patients experience from research participation may extend to their partners as well.

Previous studies have also found that research participation can give individuals a sense of reassurance due to a shared experience with fellow participants. They derived comfort and support from the knowledge that others had experienced similar problems or symptoms (Trottier et al. 2013; Grant 2015) However, in this study, some individuals reported how participating in Enroll-HD has given them a sense of shared experience with their affected parent. It provided the opportunity to talk about the condition and gave an improved sense of connectedness and understanding. Two other studies examining research participation have observed similar outcomes. Families of children with a cleft lip and palate found that participating in research had given the unaffected parents and siblings a greater appreciation of what the affected child experiences (Donoghue et al. 2014), while some participants involved in a study regarding endometriosis discussed how involvement in research provided an opportunity to talk openly about the condition within the family and brought relatives closer (Treloar et al. 2007). Taken together, these results further suggest that, particularly when regarding a genetic condition, unaffected relatives can benefit from research participation as much as patients can. However, this shared experience and improved insight could also prove quite emotionally challenging for some. Thus, the research team should be aware that participants may require additional support at times during the research process.

### Impact of participation

Another beneficial outcome of participation was an improved understanding and awareness of the condition, which has also been previously reported (Donoghue et al. 2014). Through research participation, individuals can access information that is perhaps in a more comprehensible and less intimidating format than they would find elsewhere. Indeed, several of the Enroll-HD participants relied on their assessments for most of their information and understanding as it came from a source they considered reliable. Previous work has also found that research participation can act as a coping strategy for some individuals, giving a sense of control and reducing feelings of helplessness (Trottier et al. 2013). Several of the Enroll-HD participants reported this outcome. Some of the mutation-positive participants discussed how they, and also their partners, felt better-equipped to deal with the diagnosis as a result of participation.

An interesting finding to emerge was the way in which participation in Enroll-HD led some participants to envisage “a different self”. For the mutation-positive individuals, this was an awareness of their future self and an anticipation of how things will change as they become symptomatic. For the mutation-negative individuals, it was recognition of what could have been had their 1 in 2 chances of inheriting the mutated allele resulted differently. This appeared to reinforce their feeling of being the “lucky one”, which several cited as a contributing factor in their motivations to participate in Enroll-HD. This awareness of a “different self” has not previously been observed for research participation. However, some studies have explored how predictive testing acts to reveal an underlying identity, often resulting in changes in self-perception (Armstrong et al. 1998; Klitzman 2009). Results from this study involving Enroll-HD suggest that participation in research can act similarly; reminding participants of this revealed identity or “different self”.

### Hopes and expectations

All participants were able to easily identify their hopes and expectations of Enroll-HD. Several spoke of hoping for a cure for the condition; however, most also wanted more short-term advances in symptom management. In many ways, participant expectations reflected their motivations; they were driven to participate in the hope of achieving progress and this is what they expected from the study. Participants were also overwhelmingly optimistic about the outlook for HD research and treatment. Previous studies have expressed concerns of research participants exhibiting “unrealistic optimism” (Jansen et al. 2011), often as a result of high public expectations of genetic research (Gollust et al. 2012). However, participants interviewed in this study had generally very realistic expectations of the Enroll-HD study, anticipating progress would occur slowly, over several years. Other studies have also found that participants are often keen to learn of any study progress and updates, expressing disappointment when this did not happen (Kost et al. 2011). This was an issue identified by some of the Enroll-HD participants. Despite receiving newsletters from the study, several seemed unaware of the information or described it as “scientific jargon”. However, the importance of ensuring participants are updated about progress, no matter how small, has been proven. In one study, hearing about the advances made in research was a source of hope for some participants and validated their decision to take part (Trottier et al. 2013). Furthermore, several of the Enroll-HD participants already appeared to feel directly part of the progress. Some spoke of being a jigsaw piece in a bigger picture or directly included themselves when discussing the achievements made thus far. This supports previous work suggesting that participants should be considered more as partners in research (Miller et al. 2012). To make participants aware of the progress recognises them as a necessary part of the process and respects the time and effort they are willing to contribute.

### Study limitations

Through necessity, the study was limited to English-speaking individuals, all from the North West of England, and who attend their Enroll-HD assessments in Manchester, UK. Experiences of participants recruited from other centres may be different. The sample size remained relatively small to allow for an in-depth investigation using thematic analysis, and thus, results are not meant to be generalisable to all individuals participating in Enroll-HD. The group of participants were also a relatively homogenous sample of pre-symptomatic mutation carriers or individuals not at risk. Their opinions will potentially differ to those participants of Enroll-HD who are prodromal or symptomatic as well as to those who declined research participation. Due to the interview-based approach, some could argue that pressures of social acceptability may have prevented participants from providing genuine opinions. As they have been approached by a researcher from the centre in which their Enroll-HD assessments are conducted, the urge to be polite or concerns about damaging that relationship may have prevented them from speaking freely about their experiences. However, participants shared emotional and personal family stories and appeared relaxed during the interviews, suggesting this was unlikely to be the case. To ensure this in the future, use of anonymous questionnaires or a researcher from an external centre could be considered. To allow flexibility for the participants, all of the interviews were conducted by telephone which may have also influenced patient responses and their analysis.

### Further research

A larger quantitative study is warranted with a more diverse cohort, capturing the experiences of Enroll-HD participants from other countries. As all individuals interviewed here were either unaffected or pre-symptomatic, it would also be beneficial to interview symptomatic participants or those who have declined predictive testing. A prospective longitudinal study could also assess how opinions change over time. As the participant’s partner often clearly plays an important role, it would also be interesting to explore their thoughts and experiences of the Enroll-HD process. Gaining the perspective of those who declined or dropped out of Enroll-HD participation would also provide better insight into the barriers of research participation.

### Implications for practice

Although this was a relatively small qualitative study, participants provided in-depth accounts of the factors that were important to them participating in Enroll-HD. The table below summarises key points raised by participants for researchers.

**Table:** 

Implications for practice	
Where possible, maintain the same clinical and research teams due to the benefits of the relationships with clinicians and continuity of seeing the same team at each assessment	
Acknowledge that many participants will have a lived experience of the condition and while this may encourage them to participate, will also impact their assessment experiences	
Clinicians should be aware that pre-symptomatic individuals who tested mutation positive may be anxious in the run up to their annual assessment	
Explore the support available to participants after their research visits	
Recognise the involvement and importance of partners in the research visit	
Ensure that participants are kept abreast of research progress and that the information is in a comprehensible and accessible format. Provide links to the Enroll-HD and HDBuzz websites	

## Conclusions

The findings in this study contribute to an emerging body of work on the motivations and experiences of participants in clinical research. It endeavoured to identify key themes from in-depth qualitative interviews that represent the experiences of ten individuals participating in the Enroll-HD study. As demand for human research participants increases and studies like Enroll-HD become increasingly global, research into participation provides helpful insight into why some people choose to take part and others do not. This may ultimately help to improve recruitment, engage people in research and promote long-term involvement. It is encouraging that overall, participants were very positive about their experiences in Enroll-HD and the benefits gained from participating. The results from this study also provide support for increased numbers of combined clinical and research visits for patients and their families. Participants valued the clinical information and support the Enroll-HD visits provided and relationships with the team were particularly important. The results also alluded to the potential therapeutic benefits of involvement in research, not only for the patient but also for partners and unaffected family members.
